# Non-Synonymous Polymorphisms in the *FCN1* Gene Determine Ligand-Binding Ability and Serum Levels of M-Ficolin

**DOI:** 10.1371/journal.pone.0050585

**Published:** 2012-11-28

**Authors:** Christian Gytz Ammitzbøll, Troels Rønn Kjær, Rudi Steffensen, Kristian Stengaard-Pedersen, Hans Jørgen Nielsen, Steffen Thiel, Martin Bøgsted, Jens Christian Jensenius

**Affiliations:** 1 Department of Rheumatology, Aarhus University Hospital, Aarhus, Denmark; 2 Department of Biomedicine, Aarhus University, Aarhus, Denmark; 3 Department of Clinical Immunology, Aalborg Hospital, Aarhus University Hospital, Aarhus, Denmark; 4 Department of Surgical Gastroenterology, Hvidovre University Hospital, Copenhagen, Denmark; 5 Department of Haematology, Aalborg Hospital, Aarhus University Hospital, Aarhus, Denmark; 6 Department of Mathematical Sciences, Aalborg University, Aalborg, Denmark; University of Milan, Italy

## Abstract

**Background:**

The innate immune system encompasses various recognition molecules able to sense both exogenous and endogenous danger signals arising from pathogens or damaged host cells. One such pattern-recognition molecule is M-ficolin, which is capable of activating the complement system through the lectin pathway. The lectin pathway is multifaceted with activities spanning from complement activation to coagulation, autoimmunity, ischemia-reperfusion injury and embryogenesis. Our aim was to explore associations between SNPs in *FCN1,* encoding M-ficolin and corresponding protein concentrations, and the impact of non-synonymous SNPs on protein function.

**Principal Findings:**

We genotyped 26 polymorphisms in the *FCN1* gene and found 8 of these to be associated with M-ficolin levels in a cohort of 346 blood donors. Four of those polymorphisms were located in the promoter region and exon 1 and were in high linkage disequilibrium (r^2^≥0.91). The most significant of those were the *AA* genotype of *−144C>A* (rs10117466), which was associated with an increase in M-ficolin concentration of 26% compared to the *CC* genotype. We created recombinant proteins corresponding to the five non-synonymous mutations encountered and found that the *Ser268Pro* (rs150625869) mutation lead to loss of M-ficolin production. This was backed up by clinical observations, indicating that an individual homozygote of *Ser268Pro* would be completely M-ficolin deficient. Furthermore, the *Ala218Thr* (rs148649884) and *Asn289Ser* (rs138055828) were both associated with low M-ficolin levels, and the mutations crippled the ligand-binding capability of the recombinant M-ficolin, as indicated by the low binding to Group B Streptococcus.

**Significance:**

Overall, our study interlinks the genotype and phenotype relationship concerning polymorphisms in *FCN1* and corresponding concentrations and biological functions of M-ficolin. The elucidations of these associations provide information for future genetic studies in the lectin pathway and complement system.

## Introduction

The human immune system has evolved innate and adaptive components that cooperate to protect against microbial infections while maintaining homeostasis of the body. The innate system encompasses various recognition molecules able to sense both exogenous and endogenous danger signals arising from pathogens or damaged host cells. The complement system is an important part of the innate immune system, consisting of a finely equilibrated composition of proteins. Thus it is relevant to study the influence of polymorphisms in these genes encoding the proteins, to enable the interpretation of the genotype-phenotype relationship.

**Figure 1:**
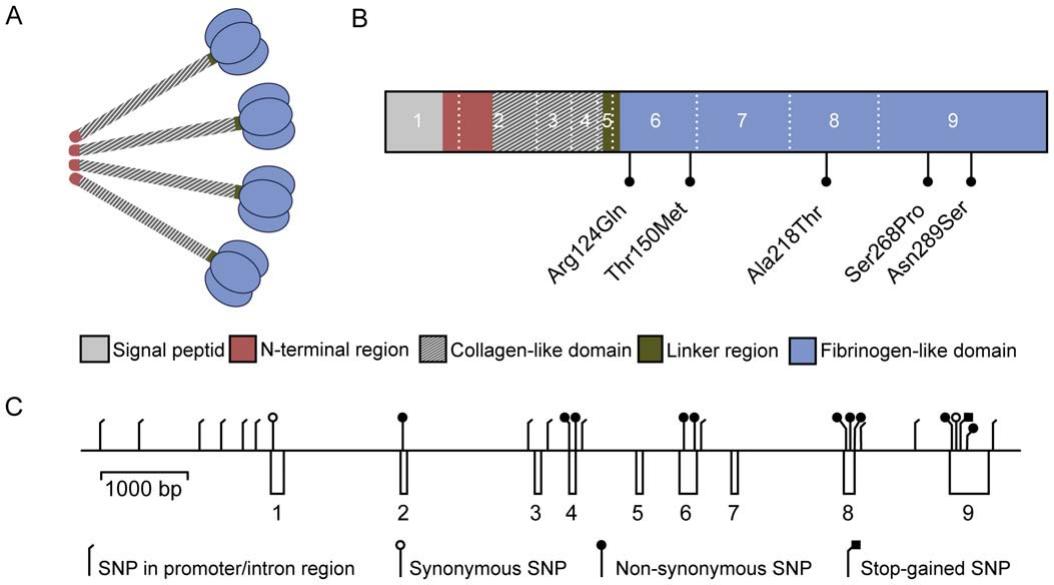
The structural and domain organization of M-ficolin and the organization of the exons in *FCN1*. A M-ficolin oligomer consisting of 4 subunits each made of 3 identical polypeptides. **B** Structure of the M-ficolin polypeptide. White numbers indicate exon and dotted line indicate exons boundaries. The 5 non-synonymous SNPs encountered in the cohort are marked. Amino acid numbers include the signal peptide of 29 residues. **C** Representation of the promoter, exon and intron region of *FCN1* drawn to scale. Exons are marked as boxes below the line and SNPs as lines above. All 26 SNPs genotyped in the cohort are marked.

The lectin pathway activates the complement system through the recognition of pathogens or altered self-structures by mannan-binding lectin (MBL) or one of the three ficolins (H-, L- and M-ficolin). The structural composition of M-ficolin is similar to that of MBL and the other ficolins, with polypeptides that trimerize into subunits, which in turn oligomerize into larger macromolecules (Fig. 1). M-ficolin form complexes with MBL-associated serine proteases (MASPs), and MASPs are converted from proenzymes to active forms when M-ficolin binds to pathogens. MASPs are then responsible for complement activation through cleavage of other complement factors. Over the past decade new knowledge broadened the role of the lectin pathway from complement activation to coagulation, autoimmunity, ischemia-reperfusion injury and embryogenesis [1]–[3].

M-ficolin is encoded by *FCN1* on chromosome 9q34, close to *FCN2* which encodes L-ficolin (Fig. 1). The two proteins show an 80% identical amino acid sequence, and phylogenetic analysis indicates that the *FCN2* gene originates from gene duplication of *FCN1* [4], [5]. The ficolins exhibit differences in tissue expression and ligand specificity, suggesting a specific role of each ficolin. H-ficolin is expressed in lung, and as for L-ficolin also in liver, whereas M-ficolin expression mainly is seen in bone marrow and peripheral leukocytes [6]. M-ficolin is synthesized by monocytes and granulocytes, secreted to the surroundings upon stimulation, but also found as a membrane associated protein on the surface of these cells [7]–[9]. Congruent with this is the correlation between the M-ficolin concentration and the number of neutrophils in the blood of healthy blood donors, pediatric cancer patients and rheumatoid arthritis patients [10], [11].

M-ficolin has marked ligand specificity for sialic acid, a property not shared with the other ficolins [12]. This feature is utilized when M-ficolin binds to capsulated bacteria, e.g., Group B Streptococcus [13]. In addition, M-ficolin binds to C-reactive protein, which enhances the binding of C-reactive protein to bacteria [14]. Clinical studies have linked M-ficolin to the occurrence of severe infections in haematological cancer undergoing chemotherapy [15] and the need for mechanical ventilation and mortality in premature infants with necrotising enterocolitis [16]. Furthermore M-ficolin is highly elevated in the synovial fluid of rheumatoid arthritis patients indicating a possible role in autoimmunity [10].

Single nucleotide polymorphisms (SNPs) in the genes of several of the lectin pathway proteins have been found to influence the corresponding concentrations in plasma [17]–[20]. Two report have appeared on associations concerning concentration of M-ficolin and SNPs in the promoter region of the *FCN1* gene, but no attempt was made to investigate for non-synonymous SNPs [21], [22].

Our main aim was to explore associations between SNPs in *FCN1* and corresponding protein concentrations in plasma. We first explored for new SNPs by sequencing the *FCN1* gene in 46 selected cases, and afterwards we analyzed 26 SNPs in the *FCN1* gene of 346 blood donors and examined for correlations to protein levels. We further created corresponding recombinant protein to 5 non-synonymous mutations and investigated for biologic function and ligand-binding capacity.

## Results

### Age and Gender Influence

**Figure 2:**
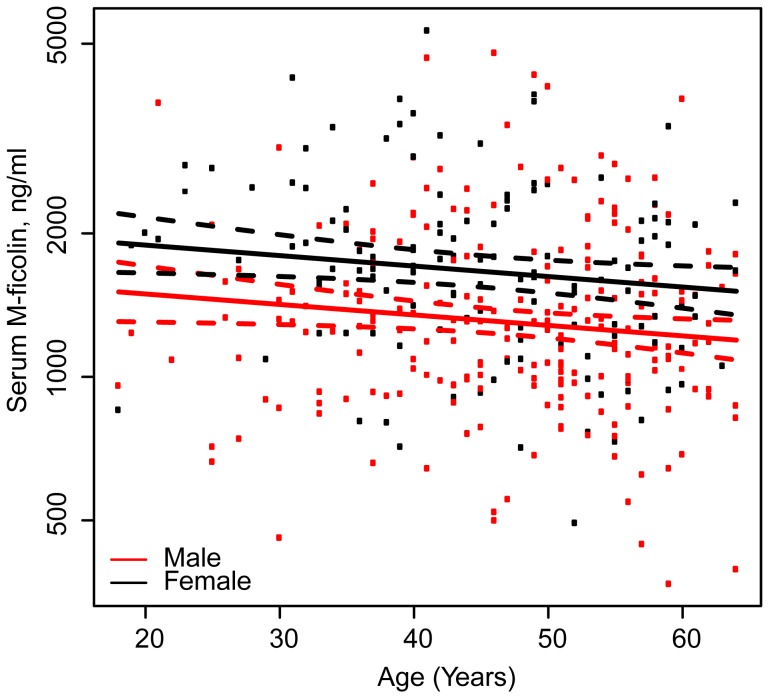
Association between age and serum concentration of M-ficolin split by gender. Full-drawn lines represents the estimated linear association for males (red) and females (black). Dotted lines represent 95% pointwise confidence intervals.

Table 1 shows blood donor characteristics, and reveals a majority of men with a median age slightly higher than the women. Prior to the SNP association analysis, the effect of age and gender on serum M-ficolin was tested using a multiple linear regression model, with serum M-ficolin as dependent variable, and age and gender as covariates. A significant association of the serum concentration of M-ficolin with gender (P<0.001) and age (P<0.03) was observed. Regarding the age-dependent decrease in the serum concentrations of M-ficolin, no significant difference was found between the genders and a linear model for the age-dependence in both genders was fitted (Fig. 2). Male gender was associated with a reduction of 21.0% (confidence interval (CI); 13.0–28.3%) and an increase in age of a decade resulted in a reduction of 5.0% (CI; 0.6–9.5%) in median M-ficolin concentration.

**Table 1:** Blood donor characteristics.

	All donors		Male		Female		P value
Number, (%)	350	(100%)	218	(62.3%)	132	(37.7%)	<0.001
Median age, (IQR)	47	(39–55)	49	(40–55)	45	(38–54)	0.021
M-ficolin, (CI)	1.43	(1.36;1.50)	1.30	(1.22;1.38)	1.67	(1.55;1.80)	<0.001

P value by Pearson’s Chi-square test for significant Male/Female ratio and Student’s t-test for age and M-ficolin concentration difference between the Male and Female population. IQR, inter quartile range. CI, 95% confidence intervals.

### SNP Exploration of *FCN1*

Twenty-eight SNPs were discovered by sequencing the promoter region and all 9 exons of the *FCN1* gene in 46 selected individuals, of which 7 at the time of sequencing were not registered with an rs-number in the dbSNP Build 133 database at the NCBI Reference Assembly (Table S1). Seven SNPs were located in the promoter region, 11 in introns, two in the 3′boundary region, and two synonymous SNPs in exons. Five of the 28 SNPs were non-synonymous causing amino acid changes (*Arg124Gln*, *Thr150Met*, *Ala218Thr*, *Ser268Pro, Asn289Ser*). All these were located in the fibrinogen-related domain of M-ficolin protein (Fig. 1B).

### Genotypes and Concentrations

Based on the above findings and the SNPs listed in the dbSNP database, 26 SNPs were chosen to be genotyped in 346 blood donors. No deviations from Hardy-Weinberg equilibrium were found for any of the genotyped SNPs (data not shown). To test the association between genotypes and serum concentrations of M-ficolin a multiple linear regression model with serum M-ficolin as dependent variable and age, gender and genotypes as well as their interactions as independent variables was applied (Table 2). From this analysis associations were observed between serum M-ficolin with age, gender and genotype, and no interaction effects on M-ficolin were observed between the independent variables gender, age and genotype.

**Table 2:** Sequential analysis of variance table for the regression model of M-ficolin versus independent variables.

	Df	SSQ	F	P value
**Main Effects**				
Age	1	1.965	11.071	0.001
Gender	1	4.406	24.834	<0.001
Genotype	26	7.513	1.623	0.032
**Interaction Effects**				
Genotype*Gender	16	2.993	1.050	0.404
Genotype*Age	17	4.031	1.331	0.173
Gender*Age	1	0.213	1.195	0.275
**Error**				
Residual	327	68.315		

(Df) degrees of freedom, (SSQ) sum of square.

Because of the effect of both gender and age on M-ficolin concentration, gender and linear age-adjustment were applied and genotypes were included one-by-one as dependent variables in a multiple linear regression analysis. Seven SNPs showed significant associations with gender and age-adjusted M-ficolin concentration; three were located in the promoter region, one synonymous in exon 1, two non-synonymous in exon 8 and 9, and one in intron 8. The age-adjusted serum M-ficolin level estimates were reported for each gender at 40 years (Table 3). The four SNPs in the promoter region and exon 1 were frequent with similar minor allele frequency around 0.35, which were in contrast to the last 3 SNPs encountered only once or twice in heterozygote state.

**Table 3:** SNPs in the FCN1 gene genotyped in 350 healthy blood donors and result of a test for their association with M-ficolin concentration, and the age-adjusted median M-ficolin concentration is given for each gender at age 40.

RS-nr	Position	Region	Aminoacid Change	Geno-type	n	Male	Female	F	P value	
						Age-adjusted M-ficolin conc. ng/ml				
rs2989727	−1981G>A	promoter		G G	52	1265 (1107;1445)	1609 (1407;1840)	1.61	0.201	
				G A	158	1313 (1206;1430)	1670 (1521;1835)			
				A A	133	1417 (1295;1550)	1803 (1624;2000)			
rs7857015	−1524T>C	promoter		T T	138	1284 (1174;1406)	1635 (1485;1800)	3.04	0.049	*
				T C	162	1346 (1239;1462)	1713 (1560;1882)			
				C C	44	1553 (1350;1787)	1977 (1700;2300)			
rs28909068	−791A>G	promoter		G A	53	1223 (1073;1394)	1554 (1361;1774)	2.87	0.091	
				A A	292	1369 (1276;1468)	1739 (1601;1889)			
rs10120023	−542G>A	promoter		G G	140	1281 (1172;1401)	1632 (1483;1795)	3.76	0.024	*
				G A	163	1347 (1240;1462)	1715 (1563;1882)			
				A A	42	1587 (1375;1831)	2021 (1734;2354)			
rs28909976	−271->insT	promoter		–	132	1419 (1297;1553)	1807 (1629;2005)	1.64	0.196	
				− insT	160	1315 (1208;1432)	1675 (1526;1838)			
				insT insT	52	1265 (1107;1445)	1611 (1409;1841)			
rs10117466	−144C>A	promoter		C C	143	1287 (1179;1406)	1626 (1478;1788)	4.24	0.015	*
				C A	161	1341 (1235;1455)	1694 (1544;1858)			
				A A	40	1619 (1398;1875)	2045 (1751;2387)			
rs10858293	33G>T	exon 1	p.Gly11Gly	T T	39	1593 (1374;1847)	2030 (1734;2375)	3.67	0.027	*
				T G	164	1348 (1242;1463)	1718 (1564;1886)			
				G G	142	1282 (1173;1402)	1634 (1486;1796)			
rs10441778	1435G>A	exon 2	p.Gly43Asp	G G	345	–	–			
rs187602432	3161G>A	intron 2		G A	4	1028 (663;1593)	1292 (825;2023)	1.51	0.219	
				G G	341	1355 (1265;1451)	1703 (1573;1844)			
rs2989722	3231C>T	intron 3		T T	139	1418 (1298;1548)	1799 (1625;1993)	1.03	0.380	
				T C	155	1317 (1209;1435)	1671 (1522;1835)			
				C C	51	1272 (1113;1454)	1615 (1410;1849)			
rs56345770	3458G>A	exon 4	p.Arg93Gln	G G	345	–	–			
rs146517825	3476G>A	exon 4	p.Arg99His	G G	345	–	–			
rs2070620	3650G>A	intron 4		G G	343	–	–			
rs147309328	4759G>A	exon 6	p.Arg124Gln	G G	345	1357(1268;1452)	1704(1574;1845)	2.77	0.097	
				G A	1	646(270;1549)	812(337;1956)			
rs56084543	4837C>T	exon 6	p.Thr150Met	C C	342	1348(1259;1443)	1697(1567;1838)	1.07	0.286	
				C T	4	1713(1102;2664)	2157(1388;3352)			
rs2070622	4888C>G	intron 6		C C	59	1326 (1168;1506)	1690(1492;1914)	1.13	0.323	
				C G	152	1311(1204;1428)	1670(1518;1838)			
				G G	126	1417(1295;1550)	1805(1627;2002)			
rs151151544	6608G>A	exon 8	p.Ser201Asn	G G	344	–	–			
rs148649884	6658G>A	exon 8	p.Ala218Thr	G A	2	594 (320;1100)	753 (406;1395)	6.90	0.009	*
				G G	342	1352 (1264;1446)	1714 (1584;1856)			
rs149439264	6727G>A	exon 8	p.Gly241Arg	G G	345	–	–			
ss522927228	6757G>A	intron 8		G G	344	1348 (1260;1443)	1692 (1563;1832)	3.95	0.048	*
				G A	1	3264 (1355;7862)	4097 (1711;9809)			
rs1888710	7554G>C	intron 8		G G	52	1279 (1121;1459)	1636 (1433;1867)	1.43	0.242	
				G C	161	1303 (1198;1417)	1666 (1519;1827)			
				C C	131	1408 (1288;1539)	1800 (1626;1994)			
rs150625869	7895T>C	exon 9	p.Ser268Pro	T T	344	1351 (1262;1445)	1701 (1572;1841)	3.43	0.065	
				T C	1	592 (247;1421)	746 (309;1799)			
rs1071583	7918A>G	exon 9	p.Gln275Gln	G G	139	1414 (1295;1545)	1807 (1633;1999)	1.94	0.146	
				G A	156	1311 (1204;1428)	1675 (1527;1838)			
				A A	48	1240 (1083;1420)	1584 (1380;1819)			
rs56094122	7929G>A	exon 9	p.Trp279Ter	G G	344	–	–			
rs138055828	7959A>G	exon 9	p.Asn289Ser	A A	345	1353 (1265;1448)	1699 (1570;1839)	6.32	0.012	*
				A G	1	443 (184;1063)	556 (230;1340)			
ss522927220	8366A>G	3' region		A G	1	592 (246;1423)	745 (308;1798)	3.42	0.065	
				A A	343	1350 (1262;1445)	1699 (1569;1840)			

Numbers in parenthesis is 95% confidence intervals.

**Figure 3:**
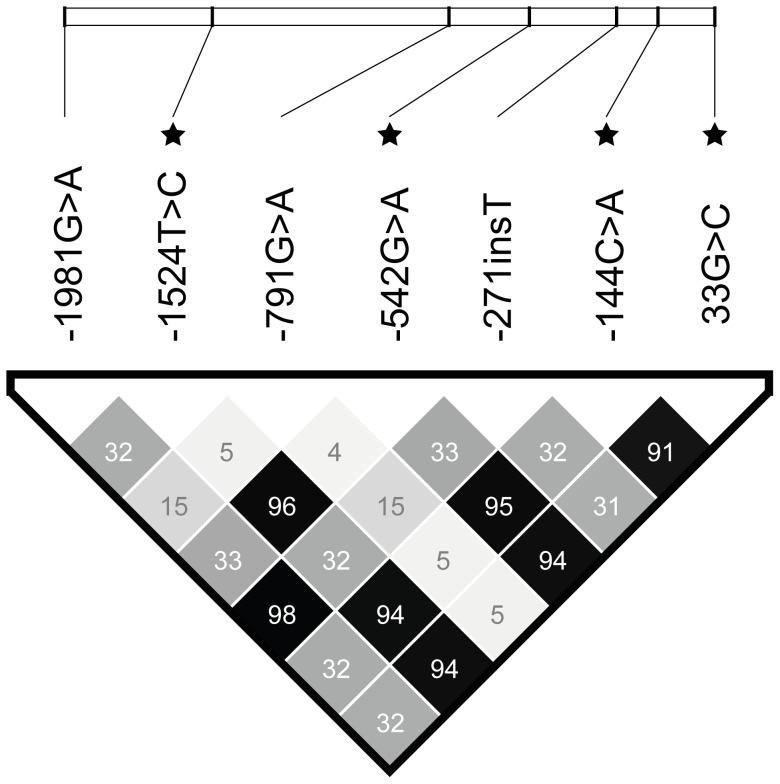
Correlation between the SNPs in the promoter region and exon 1 (R^2^ values). R^2^ is given as percent. Stars indicate SNPs that are significantly associated with M-ficolin concentration.

Linkage disequilibrium (LD) analysis revealed a very high degree of LD among the four SNPs in the promoter region and exon 1 with a significant effect on serum M-ficolin (Fig. 3). The R^2^-value between two loci, were very high between the four SNPs, with values in the range of 0.91–0.96. Since *−144C>A* had the lowest p value among the four SNPs with respect to association to serum M-ficolin (Table 2), it was used as a covariate to determine the influence of the remaining three SNPs on serum M-ficolin concentrations. None of the three SNPs contributed with further explanatory power (*−1524T>C* (P = 0.472), *−542G>A* (P = 0.428), *33G>T* (P = 0.762)) to the age-adjusted M-ficolin concentration. The minor *AA* genotype of *−144C>A* was associated with an increase of 25.8% (CI; 7.7–46.8) (P = 0.004) compared to the *CC* genotype in age-adjusted M-ficolin concentration, whereas there was no effect of the *CA* genotype compared to the *CC* genotype (P = 0.42).

Heterozygosis of *Ala218Thr*, was associated with significantly lowered age-adjusted serum concentrations of M-ficolin; the common *GG* genotype was associated with normal serum M-ficolin, and we found none with the *AA* genotype (Table 3). A similar pattern was observed with *Asn289Ser*, where heterozygosis was associated with lowered age-adjusted concentrations of M-ficolin. The *Ser268Pro* was borderline significant (P = 0.065) associated with lowered age-adjusted M-ficolin concentrations. Four individuals were heterozygote for *Thr150Met* and one for *Arg124Gln*, and none of these mutations led to significant change in age-adjusted concentrations of M-ficolin.

### Non-synonymous SNPs Discovered in the *FCN1* Gene

We genotyped 346 individuals in the search for nine non-synonymous SNPs (Fig. 1C), and five of the nine SNPs were present in a total of nine individuals. Age, gender and M-ficolin concentration of the individuals heterozygote for one of the five non-synonymous SNPs are listed in Table 4. Non-synonymous SNPs generally have high impact on phenotype. In Table 4 we report the predicted phenotypic effect of such SNPs by two computational tools; Sorting intolerant from tolerant (SIFT) [23] and polymorphism phenotyping (PolyPhen-2) [24]. SIFT is based on the premise that protein evolution is correlated with protein function. Positions important for function should be conserved in an alignment of the protein family, whereas unimportant positions should appear diverse in an alignment. The prediction of PolyPhen-2 is based on a number of features comprising the sequence, phylogenetic and structural information characterizing the substitution. Of the 5 non-synonymous SNPs found *Arg124Gln* is the only one predicted by both SIFT and PolyPhen-2 to have a benign phenotypic effect, whereas the remaining four are in varying degrees predicted to be damaging (Table 4).

**Table 4:** Frequencies, concentrations and predicted effect of non-synonymous coding SNPs found in *FCN1*.

HGVS name	Frequency of heterozygosity			M-ficolin concentration of heterozygotes in cohort (ng/ml)	SIFT^1^	PolyPhen-2^2^
	Cohort	EA^3^	AA^4^			
Arg124Gln	0.003	0.001	0.0005	643^m41^	1	benign
Thr150Met	0.012	0.007	0.003	1256^f40^,1452^m48^, 2007^f42^, 3377^m47^	0	possibly damaging
Ala218Thr	0.006	0.002	0	554^m47^, 761^f44^	0.01	probably damaging
Ser268Pro	0.003	0.005	0.0009	547^m56^	0.21	possibly damaging
Asn289Ser	0.003	0.003	0	395^m64^	0	probably damaging

**1** SIFT range from 0 to 1; a score <0.05 are predicted to be deleterious, whereas >0.05 are more likely to be tolerated.

**2** PolyPhen-2 appraises a mutation qualitatively, as benign, possibly damaging, or probably damaging. **m** male gender **f** female gender, age is given in superscript after gender. Data is derived Exome Variant Server, NHLBI Exome Sequencing Project (ESP), Seattle, WA (URL: http://evs.gs.washington.edu/EVS/) v.0.0.14. (June 20, 2012);

**3** European-American population (n = 4300).

**4** African-American population (n = 2203).

### Characterization of Recombinant Proteins Representing Non-synonymous SNPs

**Figure 4:**
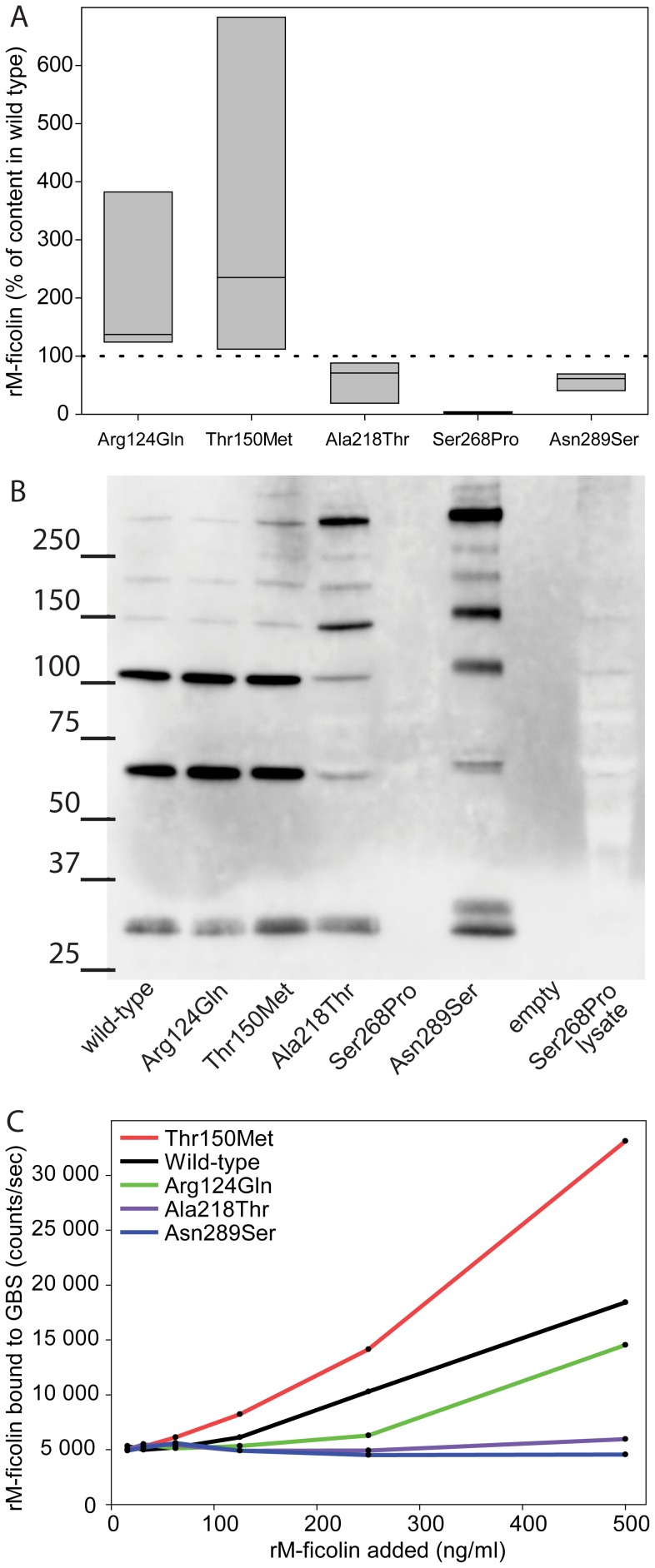
Characterization of five recombinant M-ficolin proteins. A The M-ficolin concentration measured in the supernatant of HEK293F cells transfected with plasmid encoding variants of M-ficolin. The wild type used as reference and the dotted line represents this value (100%). Boxes indicate range of data including median value. **B** Western blotting of supernatant from the wild type and the five variants of M-ficolin. For *Ser268Pro* also a lysate of the cells were used. The mutation for each variant is given beneath the lane. **C** Binding of recombinant M-ficolin to Streptococcus agalactiae serotype VI (GBS) The counts on the y-axis were obtained following incubation in GBS coated wells with recombinant M-ficolin and anti-M-ficolin antibody. Results displayed in **A** are from three while **B** and **C** are from two replicated experiments.

The effect of the amino acid change induced by the *Arg124Gln*, *Thr150Met*, *Ala218Thr*, *Ser268Pro* and *Asn289Ser* mutations were investigated *in vitro* by expression of the variants. Recombinant *Ser268Pro* was as the only protein completely immeasurable in the supernatant produced by the transfected HEK293F cells (Fig. 4A) and the cell lysate (data not shown). There was an apparent reduction in M-ficolin concentration in the supernatant of *Ala218Thr* and *Asn289Ser* transfected cells, whereas *Arg124Gln* and *Thr150Met* transfected cells had elevated levels compared to the wild-type. Western blotting using two different monoclonal anti-M-ficolin antibodies confirmed that the *Ser268Pro* recombinant protein was not expressed either in the supernatant or in the cell lysate (Fig. 4B). Ligand-binding analysis showed that both *Ala218Thr* and *Asn289Ser* were unable to recognize and bind to Group B Streptococcus, while *Arg124Gln* and *Thr150Met* bound similarly as the wild-type. The binding ability of Ser268Pro mutation was not analyzed since we were unable to produce the recombinant protein (Fig. 4C).

## Discussion

There was a substantial reduction of M-ficolin associated with male gender and aging, and these effects were independent of genetic factors. A part of the explanation for the higher M-ficolin levels in women could be the gender differences in neutrophil count, with women having 5–10% higher neutrophil counts than men [25]–[27]. Gender determined differences in neutrophil counts could directly influence the M-ficolin levels, since M-ficolin is synthesized by and associated to the circulating levels of neutrophils and monocytes in both health and disease, i.e. a higher neutrophil count is associated with higher M-ficolin level [7], [10]. The reason for the gender difference in neutrophil count is unknown, but it has been found consistently across different ethnicities. There exists also ethnic variations in neutrophil counts, most importantly with people of African origin having markedly lower neutrophil counts (15–20%), which presumably will influence the M-ficolin levels [28]. There is no decline in the neutrophil count associated with age, but most aspects of the neutrophil function are compromised in the elderly, including chemotaxis, phagocytosis, degranulation and generation of reactive oxygen species [29], [30]. One could on that basis speculate that the neutrophil in older individuals synthesizes and excretes less M-ficolin compared to the young.

We created and compared 5 recombinant M-ficolin proteins with different amino acid substitutions reflecting the non-synonymous SNPs we encountered in the population. The rare *Ser268Pro* mutation was found in one heterozygote individual showing an M-ficolin level in the lowest 2% range. The *Ser268Pro* was only borderline significant when associated to age-adjusted M-ficolin levels, and this is probably due to low statistical power. Recombinant M-ficolin containing this mutation could not be detected in the supernatant or cell lysate of transfected HEK293F cells as judged by TRIFMA and Western blotting. M-ficolin binds to its ligand in a Ca^2+^ dependent manner. *Ser268Pro* merits in that perspective special interest since Ser^268^ is predicted by the crystal structure to be an important part of the Ca^2+^ binding site through the main chain carbonyl oxygen atom of Ser^268^ [31]. Ser^268^ is furthermore close to the conserved and structurally important Cys^270^–Cys^283^ disulfide bond which stabilizes the Ca^2+^ binding site [31]. PolyPhen-2 predictions indicate a possibly damaging role while SIFT indicate a benign role of *Ser268Pro*. We hypothesize that an individual homozygote for *Ser268Pro* will be completely deficient of M-ficolin. The frequency of heterozygosity of *Ser268Pro* is listed in the databases as 0.005 (Table 4), which is similar to our findings of one heterozygote out of 345 individuals. This would translate to a calculated Hardy-Weinberg homozygote frequency of roughly one in 160.000. Up till now no one has identified a complete M-ficolin deficient individual. A known mutation likely to cause complete deficiency besides *Ser268Pro* would be the stop-mutation *Trp279Ter* (Fig. 1, Table 3), but this mutation was undetected in 4300 European-American individuals (Table S3) [32].

Both *Ala218Thr* and *Asn289Ser* have significant effects on M-ficolin levels, as they were significantly associated with the concentration of age-adjusted M-ficolin in the cohort (Table 3). The plausibility of this is supported by the predictions by both SIFT and Poly-Phred2 of the mutations to be damaging and further underpinned by in vitro studies showing a reduction compared to wild-type in recombinant M-ficolin produced by HEK293F cells.

GBS is normally recognized by M-ficolin leading to subsequent complement activation, where terminal sialic acid residues in the polysaccharide capsule on the surface of GBS is the ligand recognized by the FBG domain of M-ficolin [13]. Both *Ala218Thr* and *Asn289Ser* are located in the FBG domain of M-ficolin, and they were unable to recognize and bind to GBS (Figure 4C). The amino acid Asn^289^ is located very close to the ligand-binding pocket of M-ficolin [31], and possible changes in the tertiary protein structure induced by *Asn289Ser* would explain the impaired ligand-binding capability observed. Ala^218^ is not located in close proximity to structurally or functionally known important areas of the protein, but one could speculate that the amino acid substitutions will result in misfolding. Changes in the protein structure would render the protein susceptible to premature degradation, and hence low levels of M-ficolin. Since *Ala218Thr* and *Asn289Ser* affect both the concentration and the ligand binding ability, we speculate that an individual homozygote of one of these mutations would have a phenotype of complete deficiency.

There were four SNPs in the promoter region and exon 1 associated with M-ficolin levels. These four SNPs are all in very close linkage disequilibrium, thus further multiple regression analysis was performed to elucidate the additional effect of the three SNPs compared to the most significant SNP *−*144C>A. The three SNPs (*−*1524T>C, *−*542G>A, 33G>T) failed to add additional explanation to the model. We conclude that the four SNPs represent the same effect with respect to association with M-ficolin concentration. A recent study supports this by showing a similar association of both *−*542G>A and *−*144C>A with M-ficolin concentration in blood donors, but with a lower LD between the two (r^2^ = 0.71). This publication failed to find an association of M-ficolin levels with gender and age. The differences from the present results may be due to the lower number of samples tested [21]. The r-squared plot in this study is similar to that compiled for European-Brazilians by Boldt et al. [22]. Both studies performed in silico prediction of two of the functional FCN1 promoter polymorphisms (*−*144C>A,*−*542G>A) and found 11 transcription factors recognizing the different sequences, thereby implying a functional role of these two polymorphisms [21], [22].

When this study was planned there was no large-scale exome data available, and there was a lack of data regarding frequency for most of the SNPs reported in the databases. This lack of data prompted us to perform the exploratory sequencing for unknown SNPs. We found 5 non-synonymous SNPs that were not reported by the previously only published study on the *FCN1* gene [33]. The recently published large-scale exome data [32] allow us to evaluate if we have missed some “common” non-synonymous SNPs in our data, Table S3. There were reported 26 different non-synonymous mutations and two stop mutations encountered a total of 122 times in heterozygotic form (none were homozygotic) in 4300 European-American individuals. The five most frequent non-synonymous SNPs encountered a total of 81 times were the same five non-synonymous SNPs found in the present study. Based on this, it is likely that we have identified the majority of individuals with non-synonymous SNPs in the *FCN1* gene in the present study. Furthermore, the six non-synonymous SNPs not encountered in the Danish population in investigations are rare in the 4300 European-American individuals, as they are only encountered a total of 10 times. There are exome data for 2203 African-American individuals (not shown) and the two most frequent non-synonymous SNPs in this cohort are the *Gly43Asp* and *Arg93Gln* mutations with an allele frequency of 3.5% and 8.9%, respectively, in contrast to 0.05% for both in the European-Americans individuals [32].

The major strengths of the study was the use of exploratory sequencing of a minor group of blood donors with extreme values of M-ficolin and the use of a very robust TRIFMA assay with specific monoclonal antibody. Furthermore the creation and biological characterization of 5 recombinant proteins generated from identified non-synonymous mutations add to the translational aspects of the study.

The observed differences in M-ficolin were found in healthy individuals. It remains to be seen whether differential M-ficolin expression would be observed in individuals during acute phase reaction or various disease processes, either of which might lead to altered transcription. The present study generated new knowledge through interlinking genotype and phenotype of M-ficolin and the *FCN1* gene opening up for future genetic studies of the innate immune system in health and disease.

## Materials and Methods

### Subject and Samples

A cohort of 350 Danish blood donors aged 18–64 years was analyzed. Genomic DNA from peripheral blood leukocytes was extracted using the QIAamp DNA Mini Kit (Qiagen, Valencia, CA). Successful DNA extraction failed for 4 donors. The concentrations of M-ficolin in the sera from these patients have previously been published [34].

### Protein Measurements

M-ficolin concentrations were determined by as a time-resolved immunofluorometric assay according to the same principle as traditional enzyme-linked immunosorbent assay. In brief the M-ficolin assay is carried out as followes: diluted samples are incubated in monoclonal anti-M-ficolin antibody coated microtiter wells. Bound M-ficolin is detected by biotin-labeled monoclonal antibody followed by europium-labelled streptavidin and measurement of the bound europium by time-resolved fluorometry [34].

### Exploration of *FCN1* Polymorphisms

Genomic DNA from the individuals with the 23 highest (range 3.1–11.1 mg/l) and 23 lowest (range 0.4–0.7 mg/l) concentrations of M-ficolin was chosen for SNP exploration by DNA sequencing. The purpose of this selection was to increase the chance of finding genetic variants with a substantial impact on M-ficolin concentration. We sequenced all 9 exons, 5′- and 3′- flanking regions and 2 kb of the promoter region of *FCN1*. Sequencing was performed by Beckman Coulter Genomics, Danvers, USA. The design of PCR amplicons utilized the following criteria; a 50 bp overlap where amplicons overlapped, and at intron/exon boundaries a minimum of 50 bp of intron sequence is represented and masks dbSNP polymorphisms to avoid placing primer on SNP containing region. A test PCR reaction at a standard thermal cycling condition was performed on each amplicon using control DNA specimens, followed by sequencing. High-throughput PCR setup and sequencing included the following steps: PCR reaction setup into 384 well format plates and thermal cycling, PCR purification utilizing SPRI (solid-phase reversible immobilization), bi-directional DNA sequencing using BigDye Terminator v3.1, post reaction dye terminator removal using Agencourt CleanSEQ and sequence delineation on an ABI PRISM 3730xl with base calling and data compilation. Sequence data generated from samples were assembled along with a reference sequence, and afterwards automated polymorphism detection using Polyphred. The SNPs not encountered in the dbSNP database were submitted to NCBI.

### Genotyping

The TaqMan OpenArray genotyping system from Applied Biosystems (ABI, Foster City, CA, USA), which is a high-throughput, highly automated and relatively low-cost (per assay) system that allow testing of many SNPs in multiple individuals in parallel, was used for genotyping of 21 SNPs in the *FCN1* gene. We typed 10 SNPs with custom-designed genotyping assays and 11 SNPs with predesigned TaqMan SNPs assays (see Table S2 for assay information). OpenArray plates were manufactured by Applied Biosystems (ABI, Foster City, CA, USA). A nontemplate control (NTC) was introduced within each set of assays. TaqMan OpenArray master mix (ABI, Foster City, CA, USA) was used in this study according to the manufacture’s protocol. Samples were loaded into OpenArray plates using the OpenArray NT Autoloader and cycled using GeneAmp 9700 thermal cycler with PCR conditions according to the manufacturer’s protocol (ABI, Foster City, CA, USA). The arrays were read using the OpenArray NT Imager and the allele calls and scatter plots were generated with the genotyping software associated with the OpenArray system.

Two custom-designed SNPs (rs138055828 and rs2989722) were performed as single TaqMan assays since assay design for Open Array failed due to high CG rich region flanking these SNPs. DNA amplification was carried out in 5-µl volume containing 20 ng DNA, 0.9 µm primers and 0.2 µm probes (final concentrations), amplified in 384-well plates. PCRs were performed with the following protocol on a GeneAmp PCR 9700 (Applied Biosystems): 95°C for 10 min, followed by 40 cycles of 95°C for 15 s and 60°C for 1 min. Subsequently, end-point fluorescence was determined using the ABI PRISM 7900 HT Sequence Detection Systems and the SDS version 2.3 software (ABI, Foster City, CA, USA).

Three SNPs rs147309328, rs56084543 and rs2070622 were genotyped by sequencing performed in both directions on a 3500 Genetic Analyser (Applied Biosystems, Foster City, CA). Specific primers used to amplify the region of exon 6 and intron 6 of the *FCN1* gene were used and the fragment were amplified by an initial denaturation at 95°C for 10 min, followed by 40 cycles of 94°C for 30 s, 69°C for 30 s, 72°C for 30 s, with a final extension of 72°C for 4 min. The sequencing reactions used in this experiment were performed using the Applied Biosystems BigDye Terminator v1.1 Cycle Sequencing Kit protocol. The resulting fragment of 383 bp was analyzed with the computer software CLC Main Workbench version 6.

### Recombinant M-ficolin Variants

Expression vectors encoding M-ficolin variants with the amino acid changes *Arg124Gln*, *Thr150Met*, *Ala218Thr*, *Ser268Pro* and *Asn289Ser* were generated from a wild-type M-ficolin plasmid [35] by site-directed mutagenesis with the Quickchange II XL site-directed mutagenesis kit (cat. no. 200522, Agilent Technologies), according to the manufacturer’s instructions. Primers for site-directed mutagenesis were designed with the program primer X (http://www.bioinformatics.org/primerx/), and ordered from Eurofins MWG Operon (Ebersberg, Germany). For transient expression, plasmids were mixed with Lipofectamin-2000 (cat. no. 11668, Invitrogen) and OptiPRO SFM (cat. no. 12309, Invitrogen), and used for transfection of HEK293F cells (cat. no. R79007, Invitrogen), according to the manufacturer’s instructions. Cells were cultivated for 72 hours in Freestyle 293 Expression Medium (cat. no. 12338, Invitrogen). The supernatants were collected after centrifugation followed by concentration on centrifugation filters (Amicon Ultra 10 K, Millipore, Ballerica, MA and Vivaspin 6, Sartorius Stedim Biotech, Goettingen, Germany). The concentrated supernatants were stored at 4°C in the presence of 0.1% NaN_3_. Cells were lysed in lysisbuffer (PBS (140 mM NaCl, 8.1 mM Na_2_HPO_4_, 2.7 mM KCl, 1.5 mM KH_2_PO_4_, pH 7.4) containing 1% Triton X-100, Complete mini enzyme inhibitors (cat. no. 11 836 153 001, Roche Diagnostics, Mannheim, Germany), 1 mM PMSF (cat. no. P7626, Sigma-Aldrich) and 100 mM GlcNAc (cat. no. A8625, Sigma).

For Western blotting, concentrated supernatants and Ser268Pro transfected cell lysate were added ¼ vol SDS-PAGE sample buffer (30 mM Tris-HCL, 10% (v/v) glycerol, 8 M urea, 3% (w/v) SDS, 0.1% (w/v) bromophenol blue, pH 8.9) and electrophoresis was run on 4–12% Bis-Tris acrylamide gels (Biorad, Hercules, CA) followed by blotting onto nitrocellulose membranes (Amersham Hybond ECL, GE Healthcare, Waukesha, WI). The membranes were blocked by incubating for 1 h at room temperature in TBS (10 mM Tris- HCL, 140 mM NaCl, pH 7.4), 0.1% (v/v) NaN_3_, 0.1% (v/v) Tween-20, washed, and developed with two anti-M-ficolin antibodies (ABS 036-05 and ABS 036-01, BioPorto, Gentofte, Denmark) at 1 µg/ml primary buffer (TBS, 0.1% (v/v) NaN_3_, 0.05% Tween-20, 1 mg/ml human serum albumin (HSA), 100 µg/ml normal human immunoglobulin (nhIg) (cat. no. 007815, ZLB Behring Gmbh, Hattersheim am Main, Germany), 1 mM EDTA, pH 7.4). Subsequently, the membranes were incubated with HRP-conjugated polyclonal rabbit anti-mouse Ig antibody (cat. no. P0260, Dako, Glostrup, Denmark), diluted 1/4000 in secondary buffer (TBS, 0.05% (v/v) Tween-20, 100 µg/ml nhIg, 1 mM EDTA, pH 7.4), and developed with Supersignal West West Pico Chemiluminescent substrate (cat. no. 34080, Pierce, Rockford, IL, USA).

Binding of M-ficolin variants was tested essentially as described previously [13]. Briefly, formalin-fixed Streptococcus agalactiae serotype VI (Group B Streptococcus) was coated in microtitre plate wells at 10^8^/ml coating buffer (5 mM Na_2_CO_3_, 35 mM NaHCO_3_, 0.1% (v/v) mM NaN_3_, pH 9.6). Followed by blocking of residual bindingsites by inhibition with HSA. Dilutions of WT or mutant recombinant M-ficolin were incubated in the wells over night at 4°C, followed by wash and incubation with biotinylated anti-M-ficolin antibody and europium-labelled streptavidin.

### Statistical Analysis

M-ficolin concentrations in serum were log-normally distributed and, therefore, log-transformed before analysis. The genotype distribution was tested for deviation from Hardy-Weinberg equilibrium and the degree of linkage disequilibrium (LD) between the SNPs was estimated using the Haploview software [36]. The squared Pearson’s correlation coefficient (R^2^) was used as measure of LD between pairs of SNPs with respect to the M-ficolin protein level. Statistical analysis was performed using the statistical software system R, version 2.15.0 [37]. Student’s t-test was used to test population differences for continuous variables and Pearson’s Chi-square test for population differences for categorical variables.

Analysis of variance based on multiple linear regression models was used to investigate the association between age, gender, genotypes and M-ficolin concentrations in serum as well as individual genotypic associations with gender and age-adjusted M-ficolin concentrations in serum. Prior to SNP-wise association analysis with M-ficolin for each gender, all serum concentrations of M-ficolin were age adjusted to 40 years, using a linear model for each gender. Results with P-values below 0.05 were considered significant and throughout 95% confidence intervals are used.

### Ethics Statement

This study was approved by “The Committees on Biomedical Research Ethics of the Capital Region” (Danish: “De Videnskabsetiske Komiteer for Region Hovedstaden”). Written informed consent was obtained from all 350 blood donors that participated, and all clinical investigations were conducted according to the principles expressed in the Declaration of Helsinki.

## Supporting Information

Table S1 **SNPs exploration sequencing in the** ***FCN1*** **gene of 46 selected individuals.** All SNPs were in Hardy-Weinberg equilibrium except rs2989721, which had an observed heterozygosity of 0.125, a predicted heterozygosity of 0.492 and a Hardy-Weinberg equilibrium p value <0.001. This was most likely due to only 54.5% were genotype for this SNP. SNPs in bold were investigated further in 350 individuals. * indicate SNPs not present in the dbSNP Build 133 database at the NCBI Reference Assembly.
(DOCX)

Table S2 **Assay information for the 26 SNPs genotyped in 346 blood donors.** Data on the forward and reverse primers regarding the not custom-designed assays are not available for commercial reasons.
(DOCX)

Table S3 **Frequencies of non-synonymous and stop mutations in the** ***FCN1*** **in 4300 unrelated European-American descendants from the Exome Variant Server, NHLBI GO Exome Sequencing Project (ESP), Seattle, WA.** Data is sorted by the frequency of heterozygosity, with the most frequent at the top. The five non-synonymous SNPs found in 350 Danes are marked with red.
(DOCX)
