# Supplementary material for: Non-Synonymous Polymorphisms in the FCN1 Gene Determine Ligand-Binding Ability and Serum Levels of M-Ficolin
Source: PLoS One. 2012 Nov 28;7(11):e50585. doi: 10.1371/journal.pone.0050585 (PMC3509001; doi:10.1371/journal.pone.0050585)
Supplement: Table S1 — SNPs exploration sequencing in the FCN1 gene of 46 selected individuals. All SNPs were in Hardy-Weinberg equilibrium except rs2989721, which had an observed heterozygosity of 0.125, a predicted heterozygosity of 0.492 and a Hardy-Weinberg equilibrium p value <0.001. This was most likely due to only 54.5% were genotype for this SNP. SNPs in bold were investigated further in 350 individuals. * indicate SNPs not present in the dbSNP Build 133 database at the NCBI Reference Assembly. (DOCX) [file pone.0050585.s001.docx]

| **Region** | **rs-number** | **Position** | **Aminoacid change** | **% Genotyped** | **Minor allele frequency** |
| --- | --- | --- | --- | --- | --- |
| **promoter** | **rs2989727** | **-1981G>A** |  | **100** | **0.466** |
| **promoter** | **rs7857015** | **-1524T>C** |  | **100** | **0.295** |
| promoter | rs140024226 * | -916G>A |  | 100 | 0.023 |
| **promoter** | **rs28909068** | **-791A>G** |  | **100** | **0.125** |
| **promoter** | **rs10120023** | **-542G>A** |  | **100** | **0.295** |
| **promoter** | **rs28909976** | **-271->insT** |  | **97.7** | **0.488** |
| **promoter** | **rs10117466** | **-144C>A** |  | **97.7** | **0.279** |
| **exon1** | **rs10858293** | **33G>T** | **p.Gly11Gly** | **97.7** | **0.291** |
| **intron 3** | **rs187602432 *** | **3161G>A** |  | **100** | **0.011** |
| **intron 3** | **rs2989722** | **3231C>T** |  | **100** | **0.466** |
| intron 3 | rs3012788 | 3374G>A |  | 86.4 | 0.461 |
| intron 3 | rs2989721 | 3384T>C |  | 54.5 | 0.438 |
| **exon 6** | **rs147309328 *** | **4759G>A** | **p.Arg124Gln** | **100** | **0.011** |
| **exon 6** | **rs56084543** | **4837C>T** | **p.Thr150Met** | **100** | **0.011** |
| **intron 6** | **rs2070622** | **4888C>G** |  | **100** | **0.466** |
| intron 6 | rs2070623 | 4961G>A |  | 97.7 | 0.465 |
| exon 6 | rs2274845 | 5358T>C | p.Asn190Asn | 97.7 | 0.465 |
| **exon 8** | **rs148649884 *** | **6658G>A** | **p.Ala218Thr** | **100** | **0.011** |
| **intron 8** | **ss522927228 *** | **6757G>A** |  | **100** | **0.011** |
| intron 8 | rs2274846 | 7007T>A |  | 100 | 0.466 |
| intron 8 | rs1105324 | 7516C>T |  | 97.7 | 0.465 |
| **intron 8** | **rs1888710** | **7554G>C** |  | **97.7** | **0.465** |
| intron 8 | rs1105325 | 7566G>C |  | 97.7 | 0.465 |
| **exon 9** | **rs150625869 *** | **7895T>C** | **p.Ser268Pro** | **100** | **0.011** |
| **exon 9** | **rs1071583** | **7918A>G** | **p.Gln275Gln** | **100** | **0.477** |
| **exon 9** | **rs138055828 *** | **7959A>G** | **p.Asn289Ser** | **100** | **0.034** |
| 3´boundary | rs2989735 | 8302A>G |  | 100 | 0.477 |
| **3´boundary** | **ss522927220 *** | **8366A>G** |  | **100** | **0.011** |

**Table S1**
