# Supplementary material for: Non-Synonymous Polymorphisms in the FCN1 Gene Determine Ligand-Binding Ability and Serum Levels of M-Ficolin
Source: PLoS One. 2012 Nov 28;7(11):e50585. doi: 10.1371/journal.pone.0050585 (PMC3509001; doi:10.1371/journal.pone.0050585)
Supplement: Table S2 — Assay information for the 26 SNPs genotyped in 346 blood donors. Data on the forward and reverse primers regarding the not custom-designed assays are not available for commercial reasons. (DOCX) [file pone.0050585.s002.docx]

| **rs number** | **Assay id** | **Assay technique** | **Forward Primer** | **Reverse Primer** | **VIC Probe** | **FAM probe** |
| --- | --- | --- | --- | --- | --- | --- |
| rs10117466 | C__27830867_20 | TaqMan OpenArray |  |  |  |  |
| rs10120023 | C__27832840_10 | TaqMan OpenArray |  |  |  |  |
| rs10441778 | C__29723976_10 | TaqMan OpenArray |  |  |  |  |
| rs1071583 | C___1819018_1_ | TaqMan OpenArray |  |  |  |  |
| rs10858293 | C__25595056_10 | TaqMan OpenArray |  |  |  |  |
| rs138055828 | custom-designed assay | Single TaqMan assay | 5'-CCAGTTGATACCATTGGCATAGCT-3' | 5'-ACGCCGACTGTCATGCTT-3' | 5'-CAAACCTCAGTGGTCTC-3' | 5'-CAAACCTCAATGGTCTC-3' |
| rs146517825 | custom-designed assay | TaqMan OpenArray | 5'-CACGGGTGGCTCACCTTT-3' | 5'-CCAGGAGACCGAGGAGAGAAG-3' | 5'-TCTCCACGCATCCC-3' | 5'-CTCTCCATGCATCCC-3' |
| rs147309328 |  | Sequencing | 5'-CCGATGGCCTGGACTTTTAC-3' | 5'-TCGGGTCCGCACAGAAGC-3' | - | - |
| rs148649884 | custom-designed assay | TaqMan OpenArray | 5'-CATGCCTGGTGACAGAAAGATCT-3' | 5'-CGTCAGCCACCTTGAATGATTTG-3' | 5'-CCACCAGTTTGCTAAGT-3' | 5'-ACCACCAGTTTACTAAGT-3' |
| rs149439264 | custom-designed assay | TaqMan OpenArray | 5'-CCAGGTTCTCTCTGCTTTCCAA-3' | 5'-CTGACGAGGCAGAGAAGTACAAG-3' | 5'-ACTGCCCCCGACAAA-3' | 5'-ACTGCCCCTGACAAA-3' |
| rs150625869 | custom-designed assay | TaqMan OpenArray | 5'-TCTAACGGGCCACAACAACA-3' | 5'-ACCATTGAGGTTTGAAGCATGACA-3' | 5'-ACAATTCGAAGAACTC-3' | 5'-AATTCGGAGAACTC-3' |
| rs151151544 | custom-designed assay | TaqMan OpenArray | 5'-CTCGTCAGCCACCTTGAATGAT-3' | 5'-TCATGCCTGGTGACAGAAAGATC-3' | 5'-AGCTCGCTGCTTCCTG-3' | 5'-CTCGCTGTTTCCTG-3' |
| rs187602432 | custom-designed assay | TaqMan OpenArray | 5'-CAGCGAGGAGGCCTTGA-3' | 5'-TCTCCCTCCCTCATTTCATTACAGA-3' | 5'-AGGGACCTTGGAGCTG-3' | 5'-AGGGACCTTAGAGCTG-3' |
| rs1888710 | C___2744130_10 | TaqMan OpenArray |  |  |  |  |
| rs2070620 | C__26745030_10 | TaqMan OpenArray |  |  |  |  |
| rs2070622 |  | Sequencing | 5'-CCGATGGCCTGGACTTTTAC-3' | 5'-TCGGGTCCGCACAGAAGC-3' | - | - |
| rs28909068 | C__61859251_20 | TaqMan OpenArray |  |  |  |  |
| rs28909976 | custom-designed assay | TaqMan OpenArray | 5'-AGGTCAAGGTTTCCGAGTATTGC-3' | 5'-GTCCAGAGAGCTCCTGAGACT-3' | 5'-AAGCCGAGCTTTC-3' | 5'-CAAAGCCGATGCTTT-3' |
| rs2989722 | custom-designed assay | Single TaqMan assay | 5'-GCCTGCCACTGTCTTTCTCT-3' | 5'-CAGAGACACAGACACAGACACA-3' | 5'-CTCTGTTGCCATGTCTC-3' | 5'-TCTGTTGCCGTGTCTC-3' |
| rs2989727 | C__26745032_10 | TaqMan OpenArray |  |  |  |  |
| rs56084543 |  | Sequencing | 5'-CCGATGGCCTGGACTTTTAC-3' | 5'-TCGGGTCCGCACAGAAGC-3' | - | - |
| rs56094122 | C__89745108_10 | TaqMan OpenArray |  |  |  |  |
| rs56345770 | C__64648354_10 | TaqMan OpenArray |  |  |  |  |
| rs7857015 | custom-designed assay | TaqMan OpenArray | 5'-TGATTCCTGGCAATGGCAGTTTA-3' | 5'-CTCCTGAGAGCTTGGCAAAGT-3' | 5'-CGTCAAGCTAGCTATGTAGTA-3' | 5'-TCAAGCTAGCTGTGTAGTA-3' |
| ss522927220 | custom-designed assay | TaqMan OpenArray | 5'-TGGTTTTGATCACTTTCCTGTGTCT-3' | 5'-TGGAAAATCCTCGCAAAAGTCACT-3' | 5'-ACGGCAGGTCTTTCCCT-3' | 5'-CGGCAGGTCTTCCCCT-3' |
| ss522927228 | custom-designed assay | TaqMan OpenArray | 5'-AGGCAGAGAAGTACAAGCTGGTA-3' | 5'-CCAGGTTCTCTCTGCTTTCCAA-3' | 5'-CCACCAGCCCCAAGC-3' | 5'-CCACCAGTCCCAAGC-3' |

**Table S2**
