# Supplementary material for: Non-Synonymous Polymorphisms in the FCN1 Gene Determine Ligand-Binding Ability and Serum Levels of M-Ficolin
Source: PLoS One. 2012 Nov 28;7(11):e50585. doi: 10.1371/journal.pone.0050585 (PMC3509001; doi:10.1371/journal.pone.0050585)
Supplement: Table S3 — Frequencies of non-synonymous and stop mutations in the FCN1 in 4300 unrelated European-American descendants from the Exome Variant Server, NHLBI GO Exome Sequencing Project (ESP), Seattle, WA. Data is sorted by the frequency of heterozygosity, with the most frequent at the top. The five non-synonymous SNPs found in 350 Danes are marked with red. (DOCX) [file pone.0050585.s003.docx]

**Suplementary table 3**

| SNP Position  Chr:position | RS-number | Alleles A/B | GVS Function | Amino Acid | Protein Pos. | Genotype AA | Genotype AB | Genotype BB |
| --- | --- | --- | --- | --- | --- | --- | --- | --- |
| 9:137804881 | rs147539232 | A/G | missense | MET,THR | 150 | 0 | 32 | 4262 |
| 9:137801823 | rs150625869 | G/A | missense | PRO,SER | 268 | 0 | 22 | 4278 |
| 9:137801759 | rs138055828 | C/T | missense | SER,ASN | 289 | 0 | 14 | 4286 |
| 9:137803060 | rs148649884 | T/C | missense | THR,ALA | 218 | 0 | 7 | 4293 |
| 9:137804959 | rs147309328 | T/C | missense | GLN,ARG | 124 | 0 | 6 | 4294 |
| 9:137808283 | rs10441778 | T/C | missense | ASP,GLY | 43 | 0 | 4 | 4296 |
| 9:137806260 | rs56345770 | T/C | missense | GLN,ARG | 93 | 0 | 4 | 4296 |
| 9:137808260 | unknown | A/G | stop | stop,ARG | 51 | 0 | 2 | 4298 |
| 9:137803042 | rs147589113 | C/T | missense | GLU,LYS | 224 | 0 | 2 | 4298 |
| 9:137809689 | unknown | T/C | missense | GLN,ARG | 10 | 0 | 1 | 4299 |
| 9:137808290 | rs147292449 | C/G | missense | VAL,LEU | 41 | 0 | 1 | 4299 |
| 9:137808276 | unknown | C/G | missense | GLU,ASP | 45 | 0 | 1 | 4299 |
| 9:137808247 | rs141549661 | T/C | missense | GLU,GLY | 55 | 0 | 1 | 4299 |
| 9:137806647 | rs150223979 | T/C | missense | SER,GLY | 76 | 0 | 1 | 4299 |
| 9:137806608 | rs140975335 | A/G | missense | SER,PRO | 89 | 0 | 1 | 4299 |
| 9:137806253 | rs143361034 | G/C | missense | ASP,GLU | 95 | 0 | 1 | 4299 |
| 9:137806242 | rs146517825 | T/C | missense | HIS,ARG | 99 | 0 | 1 | 4299 |
| 9:137806243 | rs141157367 | A/G | missense | CYS,ARG | 99 | 0 | 1 | 4299 |
| 9:137806231 | unknown | A/C | stop | stop,GLY | 103 | 0 | 1 | 4299 |
| 9:137804928 | unknown | C/G | missense | MET,ILE | 134 | 0 | 1 | 4299 |
| 9:137804424 | unknown | C/T | missense | CYS,TYR | 169 | 0 | 1 | 4299 |
| 9:137803030 | rs140102154 | T/C | missense | LYS,GLU | 228 | 0 | 1 | 4299 |
| 9:137802991 | rs149439264 | T/C | missense | ARG,GLY | 241 | 0 | 1 | 4299 |
| 9:137801814 | unknown | T/C | missense | THR,ALA | 271 | 0 | 1 | 4299 |
| 9:137801809 | rs141838661 | G/C | missense | ASP,GLU | 272 | 0 | 1 | 4299 |
| 9:137801795 | rs145707204 | C/G | missense | GLY,ALA | 277 | 0 | 1 | 4299 |
| 9:137801781 | unknown | T/C | missense | ASN,ASP | 282 | 0 | 1 | 4299 |
| 9:137801654 | unknown | T/C | missense | GLN,ARG | 324 | 0 | 1 | 4299 |
|  | | | | | | | | |
